# Occurrence of marked sepsis-induced immunosuppression in pediatric septic shock: a pilot study

**DOI:** 10.1186/s13613-018-0382-x

**Published:** 2018-03-13

**Authors:** Solenn Remy, Karine Kolev-Descamps, Morgane Gossez, Fabienne Venet, Julie Demaret, Etienne Javouhey, Guillaume Monneret

**Affiliations:** 1 Hospices Civils de Lyon, Paediatric Intensive Care Unit, Mother and Children University Hospital, 59 Boulevard Pinel, 69500 Bron, France; 2 Hospices Civils de Lyon, Immunology Laboratory, E. Herriot Hospital, 69003 Lyon, France; 3 EA 7426, Pathophysiology of Injury-Induced Immunosuppression, University Claude Bernard Lyon 1, BioMérieux Hospices Civils de Lyon, E. Herriot Hospital, 69003 Lyon, France

**Keywords:** Septic shock, Immunosuppression induced, Children

## Abstract

**Background:**

While the process of sepsis-induced immunosuppression is now well described in adults, very little information is available on immune functions in pediatric sepsis. The current study investigated this in children with septic shock by performing immunomonitoring, including both innate (monocyte human leukocyte antigen-DR, mHLA-DR, expression) and adaptive immunity (lymphocyte subsets count), as well as cytokine concentrations (IL-6, IL-8, IL-10, IL-1Ra, TNF-α, IFN-γ). Subsequent objectives were to assess the associations between inflammatory response, potential immunosuppression and secondary acquired infection occurrence.

**Methods:**

Single-center prospective observational study, including children aged between 1 month and 18 years admitted to pediatric intensive care unit (PICU) for septic shock. Age-matched controls were children hospitalized for elective surgery without any infectious criteria. Blood was sampled at day 1–2, 3–5, and 7–9 after sepsis onset. mHLA-DR and lymphocyte subsets count were measured by flow cytometry and cytokine concentrations by Luminex technology.

**Results:**

A total of 26 children and 30 controls were included. Patients had lymphopenia, and mHLA-DR levels were significantly lower than controls at each time point (*p* < 0.0001). All cytokines peaked at day 1–2. Children with secondary acquired infection had lower day 3–5 mHLA-DR and higher pro-inflammatory cytokine concentrations (IL-6, IL-8 and TNF-α) at day 1–2 compared to children without secondary acquired infection.

**Conclusions:**

The higher initial inflammatory cytokine production was, the more innate immunity was altered, while evaluated by low mHLA-DR expression. Children with decreased mHLA-DR expression developed more secondary acquired infections. Upon confirmation in multicenter cohorts, these results pave the way for immunostimulation for the most immunosuppressed children in order to prevent nosocomial infections in PICU.

*Trial registration* PedIRIS study NCT02848144. Retrospectively registered 28 July 2016

**Electronic supplementary material:**

The online version of this article (10.1186/s13613-018-0382-x) contains supplementary material, which is available to authorized users.

## Background

Despite advances in critical care management, septic shock remains one of the most important causes of mortality and morbidity in children worldwide [1]. As for adults, the inability of adjunctive therapies to mitigate the deleterious effects of this condition indicates that it is likely that initial hypotheses for sepsis pathophysiology have been inadequately addressed [2]. In adults, it is now agreed that sepsis deeply perturbs immune balance by inducing a strong systemic inflammatory response and a concomitant anti-inflammatory process, acting as a negative feedback. This compensatory response may secondarily become harmful as most immune functions are compromised, and thus sepsis-induced immune alterations may play a major role in the decreased resistance to secondary acquired infections in patients who initially survive [3]. This immunosuppressive state is characterized by both abnormal innate and adaptive immune responses. In particular, patients mostly present with marked lymphopenia and decreased expression in monocyte human leukocyte antigen-DR (mHLA-DR). The latter remains, to date, the key parameter of patient monitoring and its diminished expression has been reported to be associated with increased mortality and nosocomial infection rate in adults [4–6]. In this context, and as it is already the case for cancer immunotherapy, targeted treatments aimed at rejuvenating immune responses in adult septic patients (e.g., GM-CSF, Interleukin-7, anti-PD-1/-L1) are now envisaged [7–10]. In comparison with adults, data are very scarce in children with septic shock. The impact of this altered immune response on secondary infections is poorly reported in children affected by septic shock. In order to envisage therapeutic interventions to restore immune function in children, a reliable biomarker is required to identify children at highest risk of secondary acquired infection or mortality. Such a marker is currently lacking in children. Most pediatric results are rarely specific of septic shock. They refer, as a whole, to various pediatric intensive care unit (PICU) admission causes (surgery, organ dysfunctions) [11–14], or are limited to particular cases of preterm neonates [15, 16]. Thus, the objective of the present prospective observational study was to investigate whether pediatric septic shock patients present immune alterations similar to those seen in adults. For this, immunomonitoring was performed during the first week after sepsis onset. This monitoring included mHLA-DR, lymphocyte subsets count, and phenotyping as well as measurement of plasma cytokine concentrations: interleukin-6 (IL-6), interleukin-8 (IL-8), interleukin-10 (IL-10), tumor necrosis factor-α (TNF-α), interferon-γ (IFN-γ), and interleukin-1 receptor antagonist (IL-1Ra). As control values may depend on age, and in the absence of mHLA-DR reference range in pediatrics, healthy children were also investigated to explore mHLA-DR expression during the first years of life.

## Methods

### Study population

This single-center, prospective study was held in PICU from a tertiary academic hospital (23 beds mixed medical-surgical unit, > 1100 admissions/year). Children, aged from 1 month to 18 years, were included prospectively during 24 h of PICU admission, if they presented septic shock, defined by “Surviving Sepsis Campaign” and Goldstein’s criteria [17, 18]. Exclusion criteria were: non-septic shock, chronic inflammatory disease, long-term corticosteroid treatment, transplantation and/or immunosuppressive therapy, immunodeficiency syndrome, malignant tumors. Opposition from the child and/or parent/holder of parental authority also constituted exclusion criteria. Controls were age-matched to the case group and identified among outpatients admitted for a scheduled surgery, without any criteria of infection. Exclusion criteria were the same as the case group. Three age subgroups were defined: 1 month to 2, 2–8, and > 8 years, according to the physiological age-based development of immunity in children [19].

### Clinical data and definitions

Clinical data were collected prospectively and obtained from the electronic medical record. At admission (day 1), severity of illness was evaluated using the pediatric index of mortality 2 (PIM2) [20]. Organ failure was assessed by the measurement of PEdiatric Logistic Organ Dysfunction Score, version 2 (PELOD-2) at day 1, 3, and 7 [21]. Vasoactive treatments during hospitalization in PICU were determined by the cumulative vasopressor index (CVI) [22]. We also collected incidence of secondary acquired infection, mortality (death occurring within 28 days after the onset of shock), number of mechanical ventilation-free days, and number of PICU-free days in the first 30 days. Secondary acquired infection was defined by Center for Disease Control criteria, included any new bacterial or fungal infection, distinct from initial infection, occurring more than 48 h after sepsis onset, during the first 30 days after sepsis onset [23]. Determination of secondary acquired infection was performed prospectively by one physician (SR), blinded to immunological data.

### Blood sampling for immunomonitoring

Blood samples were collected within the first 48 h after the onset of infection, again between day 3–5, and day 7–9. Samples were not available if patients were no longer in PICU. Blood was collected in EDTA tubes, transported rapidly at 4 °C to the Cellular Immunology Laboratory, and analyzed within less than 4 h. The amount of blood sample did not exceed 2.4 ml/kg, in accordance with European recommendations. At each time point, the following parameters were determined by flow cytometry: mHLA-DR, total lymphocytes and lymphocytes subpopulations (CD4^+^ and CD8^+^ T cells, natural killer [NK] cells, regulatory T cells [Treg], and B cells). In addition, after completion of cellular analysis, plasma was obtained after centrifugation and stored at − 80 °C for subsequent quantification of following circulating cytokines: IL-6, IL-8, IL-10, IL-1RA, TNF-α, and IFN-γ. For the control group, blood was sampled in the operating room immediately after induction of general anesthesia with sevoflurane, before start of surgery.

### Flow cytometry

Quantification of mHLA-DR on circulating monocytes was performed using a standardized flow cytometric assay as previously described [24.] The median fluorescence intensity of the entire monocyte population was then transformed to number of antibodies bound per cell (ABC) using calibrated PE beads (BD QuantiBRITE™ PE Beads, Becton–Dickinson San Jose, CA, USA). The following lymphocyte subsets were analyzed by flow cytometry as previously described [25, 26]: total T lymphocytes (CD45^+^ CD3^+^), CD4^+^ T lymphocytes (CD45^+^ CD4^+^ CD3^+^), CD8^+^ T lymphocytes (CD45^+^ CD8^+^ CD3^+^), total B cells (CD45^+^ CD19^+^), NK cells (CD45^+^ CD3^−^ CD56^+^), and Treg (CD4^+^ CD25^+^ CD127^−^). Results were expressed as numbers of cells per microliter of blood for lymphocyte subsets and as percentage of positive cells among total CD4^+^ lymphocyte population for Treg.

### Cytokine measurement

IL-6, IL-8, IL-10, IL-1RA, TNF-α, and IFN-γ were quantified with a single panel (Milliplex^®^ MAP Human Cytokine/Chemokine Magnetic Bead Panel, Merck Millipore) using the fluorescent bead-based multiplexed Luminex xMAP technology [27]. Analyses were performed on Bio-Plex 200 Luminex instrument using Bio-Plex software (Bio-Rad, Hercules, CA, USA).

### Statistical analysis

Results are expressed as median and interquartile range [IQR]. Comparisons between groups were analyzed using the Man–Whitney *U* test for continuous nonparametric variables; the independent paired *t* test for continuous parametric variables and the Chi-square test for categorical data. Correlation analyses were performed using the Pearson test for variables following a normal distribution, and Spearman for nonparametric variables. Kaplan–Meier analyses were performed using Youden’s index to stratify groups of patients. A *p* value < 0.05 was considered to represent significant statistical difference. Data were analyzed by using Prism6 software (GraphPad Inc., La Jolla, CA, USA).

## Results

### Subjects

**Fig. 1:**
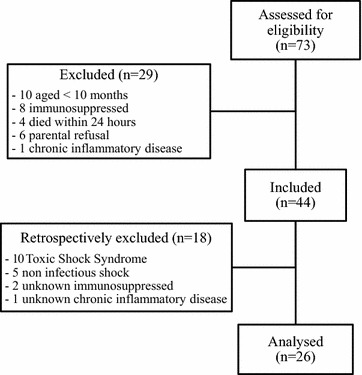
Flowchart

Between September 2014 and July 2016, 73 children were screened for septic shock. Among these, 47 were excluded (toxic shock syndrome, non-infectious shock, known immunosuppression, or chronic inflammatory disease). A total of 26 children were included and analyzed (Fig. 1). Demographic data are presented in Table 1. Nine children (35%) presented a complex chronic condition. The most frequent was prematurity (*n* = 4), but all children presented a corrected age greater than 1 month. Others presented psychomotor delay (*n* = 2), polymalformative disease (*n* = 2), and sickle cell disease (*n* = 1). Two children died: one precociously at day 2, due to refractory shock with multi-organ failure; and the second one at day 32, with withdrawal treatment for major brain damage after meningitis. A total of 30 healthy children were included as controls. They were age-matched with children in septic shock (*p* = 0.3, Mann–Whitney): 11 were aged between 1 month and 2 years, 14 between 2 and 8 years, and 5 > 8 years.

**Table 1:** Characteristics of the 26 children with septic shock included in the study

	Septic shock (*n* = 26)
Age (years)	2.12 [0.47–4.60]
Male gender [*n* (%)]	11 (42)
Complex chronic conditions [*n* (%)]	9 (35)
Primary nosocomial infection	1 (3.9)
Site of initial infection [*n* (%)]	
Blood	3 (11.5)
Lung	4 (15.4)
Abdomen	8 (30.8)
Multi-site	9 (34.6)
Others	1 (3.9)
No documentation	1 (3.9)
Microbiology	
Gram-negative bacteria	
*Neisseria meningitidis*	8 (30.8)
*Escherichia coli*	3 (11.5)
Klebsiella species	2 (7.7)
Enterobacter species	2 (7.7)
*Campylobacter jejuni*	1 (3.8)
*Haemophilus influenzae*	1 (3.8)
Gram-positive bacteria	
*Streptococcus pneumoniae*	3 (11.5)
*Streptococcus pyogenes*	2 (7.7)
*Staphylococcus aureus*	1 (3.8)
Viruses	
*Parainfluenzae virus*	1 (3.8)
PIM2 admission (%)	8.1 [3.2–17.4]
PELOD-2	
Day 1	9.5 [2.75–12.0]
Day 3	4.0 [0.0–10.0]
Day 7	0.0 [0.0–5.0]
CVI	4.0 [0.0–7.0]
ICU-free days in 30 days	23.0 [19.0–26.0]
Secondary acquired infections [*n* (%)]	8 (30)
Mortality [*n* (%)]	2 (7)

Values are expressed as median [interquartile range], or a number (percentage)

*PIM2* pediatric index of mortality 2, *PELOD-2* PEdiatric Logistic Organ Dysfunction score, version 2, *CVI* cumulative vasopressor index

### mHLA-DR expression

**Fig. 2:**
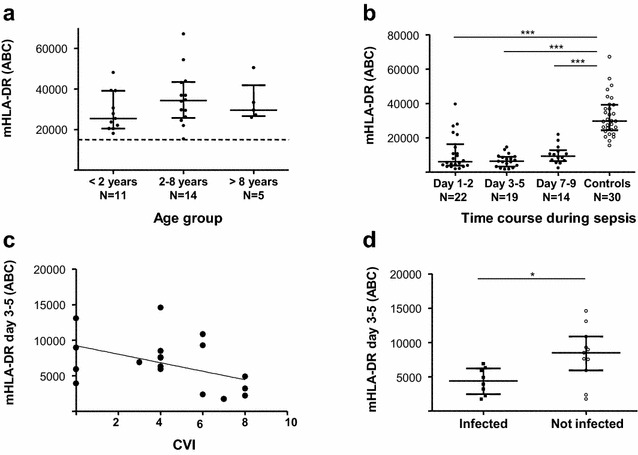
mHLA-DR measurements in pediatric septic shock. **a** mHLA-DR expression in healthy children: no difference according to age groups (*p* = 0.35; ANOVA). Dashed line depicts usual threshold to define normal values in adults. **b** mHLA-DR was significantly decreased at each time point during septic shock, than controls (*p* < 0.001; Mann–Whitney). **c** mHLA-DR at day 3–5 was significantly negatively correlated with cumulative vasopressor index, CVI (*r* = − 0.50; *p* = 0.031; Spearman). **d** mHLA-DR was significantly lower in children with secondary acquired infection than those without (*p* = 0.022; Student *t* test)

In healthy control children, mHLA-DR was > 25,000 ABC; among those aged between 1 month to 2 years it was (median [IQR]) 25,477 ABC [20,478–39,143], among those aged 2–8 years it was 34,295 ABC [25,763–43,368], and among those older than 8 years it was 29,597 ABC [26,636–41,939]; there was no significant difference between age subgroups (*p* = 0.35; ANOVA, Fig. 2a). There was also no correlation between age and mHLA-DR (*r* = 0.018).

In children with septic shock, mHLA-DR was 6066 ABC [3737–16,310] at day 1–2, 6308 ABC [3185–8965] at day 3–5, and 9323 ABC [6384–12,738] at 7–9; at each time point values were significantly different to that found in healthy control children (*p* < 0.0001; Fig. 2b). There was no correlation between mHLA-DR and PIM2 or PELOD-2 scores, at any time point, while a significant correlation was found between day 3–5 mHLA-DR and CVI (*r* = − 0.50; *p* = 0.031; Fig. 2c).

### Cytokines

For all cytokines, maximal elevated values were measured on early time point. Then, they presented a gradual decrease on following days but remained significantly elevated in comparison with normal values (Table 2). At day 1–2, IL-6 and IL-8 levels were significantly correlated with PELOD-2 (*r* = 0.52, *p* = 0.012 and *r* = 0.43, *p* = 0.048, respectively) and with CVI (*r* = 0.65, *p* = 0.001 and *r* = 0.51, *p* = 0.015, respectively). No correlation between initial cytokines concentrations and PIM2 score was found. Also, there was no correlation between initial cytokines levels and mHLA-DR at day 1–2. However, IL-8, IL-10, IL-1ra and TNF-α levels at day 1–2 were significantly negatively correlated with mHLA-DR at day 3–5 (*r* = − 0.84, *p* < 0.0001; *r* = − 0.59, *p* = 0.0198; *r* = − 0.58, *p* = 0.023; *r* = − 0.69, *p* = 0.0042, respectively).

**Table 2:** Plasma cytokines levels from healthy children and children with septic shock

	Healthy controls	Children with septic shock		
		Day 1–2	Day 3–5	Day 7–9
IL-6	0.0 [0.0–0.36]	178.5 [25.77–3311]	18.13 [12.92–172.5]	17.97 [3.30–33.08]
IL-8	2.05 [0.43–5.06]	51.72 [19.07–145.7]	21.45 [11.42–56.36]	15.00 [7.98–37.98]
IL-10	0.36 [0.0–4.44]	43.37 [17.11–331.6]	8.26 [3.44–21.66]	11.83 [1.70–23.73]
IL1-RA	0.0 [0.0–17.16]	136.9 [23.72–647.8]	48.02 [0.0–153.2]	56.54 [0.0–121.3]
TNF-α	4.91 [3.35–7.11]	25.13 [14.52–40.47]	8.37 [5.34–14.62]	10.15 [4.10–15.43]
INF-γ	4.98 [1.82–6.45]	15.81 [6.55–24.22]	8.07 [2.78–11.45]	10.92 [4.65–23.70]

Values (pg/ml) are expressed as median [IQR]. All cytokine values in children with septic shock at all-time points were different from those of healthy controls

### Lymphocyte subsets

**Fig. 3:**
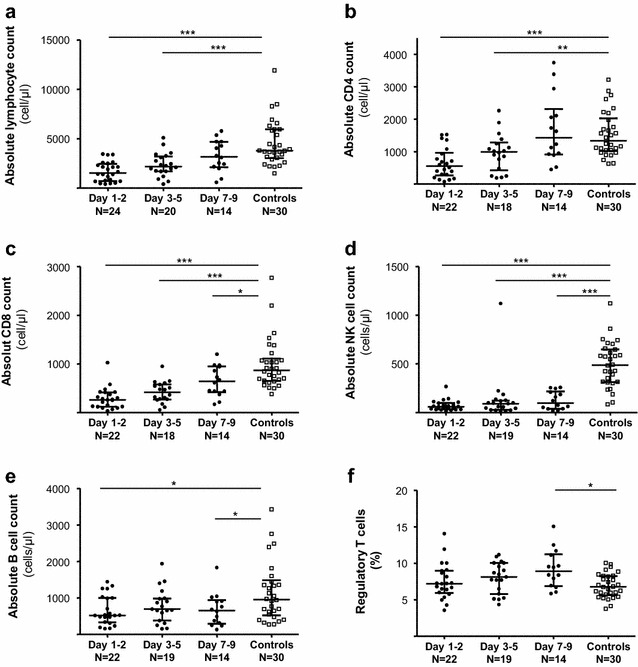
Time course of lymphocytes’ subsets during septic shock. **a** Total lymphocytes, **b** CD4^+^ T cells, **c** CD8^+^ T cells, **d** NK cells, **e** B cells (from **a** to **e**, results as cell number/µl), **f** proportion of regulatory T cells (among CD4 + lymphocytes). *p < 0.05; **p < 0.01; ***p < 0.0001

Lymphocyte subsets from healthy control children are presented in Table 3. Among septic patients the total lymphocyte count was significantly lower than in healthy control children at day 1–2 and day 3–5; there was no significant difference at day 7–9 (Fig. 3a). Similar results were observed for CD4^+^ (Fig. 3b) and CD8^+^ T cell (Fig. 3c) subsets. The median total NK cell count was significantly lower as compared to healthy control children over the whole monitoring period (Fig. 3d). In contrast and, although diminished, B cells were modestly affected (Fig. 3e). The median proportion of Treg was initially similar to that found in healthy control children; this increased at day 3–5, and became significantly greater at day 7–9 (Fig. 3f). Of note, we did not find any correlation between lymphocytes subsets and CVI (data not shown).

**Table 3:** mHLA-DR and lymphocytes subsets depending on age in healthy and septic children

	0–2 years		2–8 years		> 8 years	
	Healthy (*n* = 11)	Septic (*n* = 11)	Healthy (*n* = 14)	Septic (*n* = 6)	Healthy (*n* = 5)	Septic (*n* = 2)
mHLA-DR (ABC)	25,477 [20,478–39,143]	6302 [2187–9278]	34,295 [25,763–43,368]	5913 [2977–8911]	29,597 [26,636–41,939]	7927 [6888–8965]
Total lymphocytes (absolute count; cells/µl)	5934 [4148–8572]	2736 [1962–3550]	3623 [2940–4358]	1680 [897–2453]	2374 [1831–3298]	1047 [403–1691]
CD4^+^ T cells (absolute count; cells/µl)	2187 [1566–2738]	1186 [939–1640]	1184 [968–1583]	574 [236–478]	895 [682–1100]	332 [187–478]
CD8^+^ T cells (absolute count; cells/µl)	1033 [910–1380]	487 [311–591]	758 [631–1264]	365 [155–608]	689 [467–924]	161 [51–270]
NK cells (absolute count; cells/µl)	645 [392–753]	58 [25–124]	424 [295–576]	96 [35–156]	315 [191–645]	60 [4–117]
B cells (absolute count; cells/µl)	1811 [1326–2750]	901 [648–1397]	729 [563–988]	622 [288–791]	352 [273–508]	422 [150–693]
Regulatory T cells (% among CD4^+^)	7.67 [5.39–8.24]	7.72 [5.1–9.0]	6.29 [5.29–8.31]	8.83 [7.1–10.3]	6.80 [6.10–8.48]	7.9 [5.8–10.1]

Values are expressed as median [IQR] according to age group. Septic children values were obtained at day 3

### Secondary acquired infections

A secondary acquired infection was diagnosed in eight children (Additional file 1: Tables S1 and S2). The median time to onset of these infections was 13.5 days (range: [9–25]). Compared to children who did not developed secondary acquired infection, the sole difference was a longer length of hospital stay for children who presented secondary acquired infection (*p* = 0.009; Additional file 1: Table S1). Between these two groups, no difference was observed concerning duration of invasive equipment, PICU staying, complex chronic condition and severity scores. No significant difference in lymphocyte subset was observed between children with or without secondary acquired infections. No association was found between persistent lymphopenia (defined as an absolute lymphocyte count of < 1000 cells/µl for 3 days) and development of secondary acquired infection. At day 1–2, there was no significant difference in mHLA-DR values; at day 3–5 those with secondary acquired infection had a significantly lower level of mHLA-DR (4398 ABC [2437–6212]) than those without (8474 ABC [5904–10,844], *p* = 0.022; Fig. 2d). At day 1–2, children with secondary acquired infection had higher concentrations of IL-6, IL-8, and TNF-ɑ than those without (Additional file 1: Table S1). There was no significant difference in cytokine concentrations at other time points. The area under the curve from receiver operating curve (ROC) analysis for the risk to develop a secondary acquired infection were all significantly > 0.8 for these four significant parameters (mHLA-DR at day 3–5, and IL-6, IL-8 and TNF-ɑ at day 1–2, Additional file 1: Table S3). In line with this, mHLA-DR at day 3–5 was negatively correlated with cytokines (except IFN-γ). Owing to the sample size, no multivariate regression could be performed to test independence between mHLA-DR and cytokines for the prediction of risk of secondary acquired infection occurrence.

We next performed Kaplan–Meier analyses associating mHLA-DR values and each cytokine level separately, which found that children with lowest values of mHLA-DR and highest cytokine levels were significantly more likely to be infected. Those with mHLA-DR above the threshold remained uninfected, independently of cytokine level (Additional file 1: Fig. S1).

## Discussion

The present investigation is, to the best of our knowledge, the first prospective pediatric study reporting a wide immune monitoring, specifically in septic shock. However, the first important result of this study is the values of mHLA-DR expression in healthy children. There was no variation according to age (between 6 months and 17 years) and, furthermore, the values are not different to those reported in adults [28]. These results strongly suggest that there is no difference between healthy adults and children in terms of mHLA-DR expression. Important fall in mHLA-DR during pediatric septic shock seems to be similar to those observed during adult septic shock.

The cytokine storm reported herein, involving both pro and anti-inflammatory cytokines, was found in the initial period after shock (day 1–2). At the same time, children who later developed a secondary acquired infection had higher plasma cytokine concentrations (i.e., IL-6, IL-8, and TNF-α). This is in agreement with observations from genomic studies that have found that early mRNA expression modulations of cytokines and apoptotic genes were associated with deleterious outcomes (mortality, secondary infections) [29, 30]. Moreover, to the best of our knowledge, this is the first pediatric study which reported correlation between initial cytokine storm and alteration of innate immunity, represented by loss of mHLA-DR.

Concerning adaptive immunity, B and T lymphocytes were diminished initially, but less impacted and corrected faster than mHLA-DR alteration. The modest diminution of B cells constitutes a difference with that found in adults, for instance Monserrat et al. [31] described severe abnormality of circulating B lymphocytes associated with mortality. Concerning NK cells, Halstead et al. [32] observed a decrease at the beginning of sepsis in children. The present study provides supplementary data since a deep and prolonged alteration of circulating NK cells was found. Although we did not explore this side, NK cell alterations could promote infections by opportunistic viral pathogens [33]. In contrast with the study reported by Muszynski et al. [14], an increase in Treg proportion during the first week was observed herein. This kinetic seems similar to that described in adult septic shock, where an increased proportion of Treg was associated with poor outcome [34]. Here, as for other lymphocyte parameters, no association with nosocomial infections was found. Additional functional testing (proliferation, intracellular cytokine production) would be likely informative and deserves to be further investigated. At this stage, the present lymphocyte data seem to indicate that altered lymphocyte count rapidly self-resolves, in contrast with observations made in adults.

With regards to innate immunity, loss of mHLA-DR has emerged as a gold standard biomarker owing to its association with altered monocyte functionality, increased mortality, and nosocomial infection rate after adult septic shock [6, 35]. In accordance, we report here a significant fall in mHLA-DR in children. Importantly, the lowest values at day 3–5 were found in patients who developed secondary acquired infections. At this time point, all patients with forthcoming infections presented mHLA-DR below 8000 ABC (i.e., the usual threshold for defining the most severely immunosuppressed adult patients [36]). Most pediatric studies have used ex vivo LPS-induced TNF-α production by monocytes to assess innate immunity function. However, Drewry et al. [35] presented recently mHLA-DR as a better predictor of deleterious outcomes than LPS-stimulated TNF-α production. Add to our results, these data reinforced the idea to use mHLA-DR as biomarker of altered innate immunity in pediatric studies. Due to low number of deaths in the present cohort, we did not investigate the potential association with mortality. Although not all obtained using a standardized measurement protocol, the pediatric mHLA-DR data available in the literature are in agreement with that reported herein. For example, Manzoli et al. [13] found the extent of mHLA-DR level reduction during the first week after sepsis onset to be associated with mortality (23% mortality), and Genel et al. [16] also reported lower levels of mHLA-DR among infected neonates who did not survive compared to those who did (20% mortality). Decreased mHLA-DR has also been described in pediatric surgery and was associated with later sepsis and pneumonia [37, 38]. In addition, Hall et al. [11] reported that, in children with multiple organ dysfunction syndrome, persistent decreased TNF-α release (that reflects monocyte functionality) over 5 days was associated with development of secondary infection. In addition, some in vitro and animal studies suggest potential role of norepinephrine in the development of sepsis-induced immunosuppression [39]. These data reinforced our significant correlation between day 3-5 mHLA-DR and CVI.

Collectively, this indicates that, as in adults, mHLA-DR presents potential for the identification of the most severe immunosuppressed children. The next step would, of course, be to perform multivariate analysis in a larger patient sample to explore the independence of each parameter in predicting deleterious outcomes. That given, Kaplan–Meier analyses found that although different markers (i.e., day 3–5 mHLA-DR and early elevated cytokines) were associated with secondary acquired infections, the weight of mHLA-DR was more important. Interestingly, as observed in adult trauma patients, association between early cytokines production and low day 3–5 mHLA-DR appeared as the poorest scenario in pediatric septic shock [40].

Furthermore, secondary acquired infections represent a major economic burden by significantly extending length of hospital stay [41, 42], and concordantly herein the length of hospitalization in those with secondary infection doubled (and this difference was significant). Taken together, these data reinforce the idea that most immunosuppressed septic children might benefit from immunostimulation as an adjunctive therapy [43]. Clinical trials are in progress with GM-CSF (NCT02361528) or IL-7 (NCT02640807/NCT02797431) in adults, and standardized tools are currently used to stratify patients in those trials. This progressively paves the way for this kind of approach in pediatrics, especially for innate immunity that seems to be more affected.

The study does, however, have some limitations. First, the relatively small number of included children precluded multivariate analyses to be performed. Second, some children were discharged from PICU before all samples were obtained, and therefore the time course of different immune parameters in less severe children was not determined. Due to the relatively short follow-up period, we cannot conclude on the long-term outcome of these children treated for septic shock. In addition, functional testing should be performed in next studies. Theses aspects need to be further explored, and ideally in a multicenter study.

## Conclusion

As in adults, septic shock in pediatric patients induced marked alterations in immune parameters in accordance with the occurrence of a state of immunosuppression. The present results, in particular the association between low mHLA-DR expression and deleterious outcomes, deserves to be assessed and confirmed in multicenter studies.

## Additional file

**Additional file 1.** Online data supplement.

## Abbreviations

ABC: antibodies bound per cell
CVI: cumulative vasopressor index
IFN-γ: interferon-γ
IL-1Ra: interleukine-1 receptor antagonist
IL-6: interleukine-6
IL-8: interleukine-8
IL-10: interleukine-10
mHLA-DR: monocyte human leukocyte antigen-DR
NK cells: natural killer cells
PELOD-2: PEdiatric Logistic Organ Dysfunction Score, version 2
PICU: pediatric intensive care unit
PIM2: pediatric index of mortality 2
TNF-ɑ: tumor necrosis factor-*α*
Treg: regulatory T cells
