# Supplementary material for: Occurrence of marked sepsis-induced immunosuppression in pediatric septic shock: a pilot study
Source: Ann Intensive Care. 2018 Mar 13;8:36. doi: 10.1186/s13613-018-0382-x (PMC5849582; doi:10.1186/s13613-018-0382-x)
Supplement: Supplementary file 1 — Additional file 1. Online data supplement. [file 13613_2018_382_MOESM1_ESM.docx]

**Annals of Intensive Care**

**Occurrence of marked sepsis-induced immunosuppression in paediatric septic shock**

Solenn REMY ¹, Karine KOLEV-DESCAMPS ¹, Morgane GOSSEZ ², Fabienne VENET ² ³, Julie DEMARET ², Etienne JAVOUHEY ¹, Guillaume MONNERET ² ³

**Affiliations:**

¹ Hospices Civils de Lyon, Paediatric Intensive Care Unit, Mother and Children University Hospital, 59 Boulevard Pinel, 69500 Bron, France

² Hospices Civils de Lyon, Immunology Laboratory, E. Herriot Hospital, Lyon, 69003, France

³ EA 7426, Pathophysiology of injury-induced immunosuppression, University Claude Bernard Lyon 1, BioMérieux Hospices Civils de Lyon, E. Herriot Hospital, Lyon, 69003, France

**Corresponding author:**

Guillaume MONNERET Cellular Immunology Laboratory, Hôpital E. Herriot – Hospices Civils de Lyon, France Pavillon E – 5 place d’Arsonval 69437 LYON Cedex 03 – France guillaume.monneret@chu-lyon.fr Tel: +33 4 72 11 97 58

Table S1: Comparison of main characteristics and immune monitoring measures in children with and without secondary acquired infection

|  | **Secondary acquired infection**  **(n = 8)** | **No secondary acquired infection**  **(n = 17)** | ***p*** |
| --- | --- | --- | --- |
| Age (years) | 1.4 [0.6-5.7] | 2.6 [0.5-4.3] | 1.0 |
| Male gender, n (%) | 3 (38) | 8 (47) | 1.0 |
| Complex chronic condition,  n (%) | 3 (38) | 5 (29) | 1.0 |
| PICU stay (duration, days) | 7.0 [4.5-31.25] | 4.0 [3.0-10.0] | 0.11 |
| Length of hospital stay (days) | 33.5 [19.5-58.0] | 14.0 [7.5-21.0] | **0.009** |
| Duration of invasive equipment (days) |  |  |  |
| Intubation | 5.5 [0.0-19.0] | 2.0 [0.0-4.5] | 0.20 |
| Central Venous Catheter | 5.0 [3.0-9.0] | 5.0 [0.0-10.5] | 0.98 |
| PIM2 | 10.4 [3.0-17.1] | 8.0 [3.1-18.8] | 0.79 |
| PELOD-2 |  |  |  |
| Day 1 | 10.5 [5.75-13.5] | 7.0 [2.0-11.5] | 0.36 |
| Day 3 | 9.0 [3.25-13.5] | 3.0 [0.0-6.0] | 0.06 |
| Day 7 | 0.0 [0.0-6.0] | 0.0 [0.0-4.25] | 0.79 |
| CVI | 5.5 [3.25-8.0] | 4.0 [0.0-6.0] | 0.16 |
| Mortality, n (%) | 1 (13) | 0 | 0.32 |
| mHLA-DR D1-2 (ABC) | 4,146 [3,189-21,841] | 8,704 [4,321-16,621] | 0.28 |
| mHLA-DR D3-5 (ABC) | 4,398 [2,437-6,212] | 8,474 [5,904-10,844] | **0.022** |
| IL-6 D1-2 (pg/ml) | 976.4 [179.2-15,966] | 44.78 [18.23-224.7] | **0.028** |
| IL-8 D1-2 (pg/ml) | 129.1 [54.99-1,330] | 27.91 [10.81-67.68] | **0.019** |
| IL-10 D1-2 (pg/ml) | 53.98 [37.94-1,014] | 28.04 [6.21-88.0] | 0.080 |
| IL-1ra D1-2 (pg/ml) | 203.6 [85.30-3,850] | 125.4 [12.0-270.7] | 0.11 |
| TNF-α D1-2 (pg/ml) | 40.04 [23.35-135.6] | 16.14 [7.93-27.64] | **0.023** |
| IFN-γ D1-2 (pg/ml) | 11.45 [7.38-15.47] | 19.22 [3.96-25.84] | 0.74 |
|  |  |  |  |
|  | | | |

Values are expressed as median [25th–75th percentiles, IQR], or a number (percentage). One child died early at day 2, so was excluded from this analysis. ABC: antibody bound per cell; CVI: Cumulative Vasopressor Index; D: day, PELOD-2: PEdiatric Logistic Organ Dysfunction Score, version 2, PICU: pediatric intensive care unit, PIM2: Pediatric Index of Mortality 2.

**Table S2: Patients’ characteristics with secondary acquired infections**

| **Age**  **(years)** | **Complex Chronic**  **Condition** | **Initial**  **Infection** | **Initial**  **bacterium** | **Secondary**  **infection** | **Secondary bacterium** | **Time to**  **onset**  **(days)** |
| --- | --- | --- | --- | --- | --- | --- |
| 1.4 | - | Purpura Fulminans | *Neisseria meningitidis* | Cutaneous superinfection (necrotic purpuric) | *Staphylococcus aureus* | 25 |
| 5 | Short bowel syndrome | Septicemia on CVC | *Enterobacter cloaque* | CVC infection recurrence | *Staphylococcus aureus* | 22 |
| 1.4 | - | Purpura Fulminans | *Neisseria meningitidis* | Multifocal osteitis | Not documented | 9 |
| 13.4 | - | Peritonitis | *Escherichia coli*  *Streptococcus anginosus* | Peritoneal abscess | *Streptococcus anginosus*  *Bacteroides fragilis* | 17 |
| 0.5 | - | Meningitis with septicemia | *Streptococcus pneumoniae* | Ventilator-associated pneumonia  Pyelonephritis | *Pseudomonas aeruginosa*  *Escherichia coli* | 10 |
| 0.2 | - | Meningitis with septicemia | *Streptococcus pneumoniae* | Empyema  Bronchiolitis | Not documented  Respiratory Syncytial Virus | 12 |
| 1.1 | Sickle Cell Disease | Meningitis with septicemia | *Streptococcus pneumoniae* | Osteomyelitis | Not documented | 15 |
| 6 | Partial  Trisomy 3 | Pyelonephritis complicated by mesenteric ischemia | *Escherichia coli* | Peritonitis | *Candida albicans* | 10 |
|  |  | | | | | |

Despite the lack of bacteriological documentation, 2 patients were considered with secondary infection. One patient with multifocal osteitis after purpura fulminans, considered as a complication from cutaneous superinfection, according to MRI data. Second patient presented clinical ankle osteomyelitis, confirmed by MRI, 15 days after pneumococcal meningitis, but after 10 days without infectious symptom (neither fever nor biological inflammatory syndrome).

Table S3: Receiver operating curve analysis of mHLA-DR at day 3-5, IL-6, IL-8, TNF-α levels at day 1-2, concerning children with and without secondary acquired infection.

|  | **AUC** | **Youden’s index** | ***p*** |
| --- | --- | --- | --- |
| **mHLA-DR D3-5** | 0.818 | 7,205 ABC | 0.021 |
| **IL-6 D1-2** | 0.806 | 178.5 pg/ml | 0.025 |
| **IL-8 D1-2** | 0.827 | 51.72 pg/ml | 0.017 |
| **TNF-α D1-2** | 0.816 | 33.18 pg/ml | 0.021 |

AUC: Area under curve


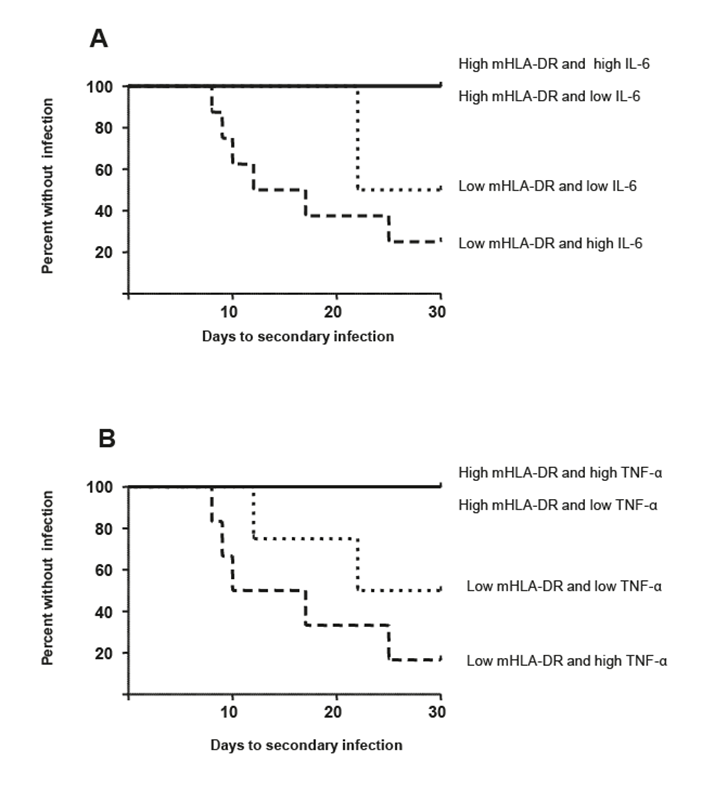


**Figure S1 :** **Kaplan-Meier analyses for secondary acquired infection**

A: mHLA-DR at day 3-5 and IL-6 at day 1-2. B: mHLA-DR at day 3-5 and TNF-α at day 1-2. For each parameter, children were dichotomized as < (low) or > (high) to Youden indexes as established in table 6 (mHLA-DR and IL-8 did not provide significant information).
